# Biological Functions and Molecular Mechanisms of Antibiotic Tigecycline in the Treatment of Cancers

**DOI:** 10.3390/ijms20143577

**Published:** 2019-07-22

**Authors:** Zhen Dong, Muhammad Nadeem Abbas, Saima Kausar, Jie Yang, Lin Li, Li Tan, Hongjuan Cui

**Affiliations:** 1 State Key Laboratory of Silkworm Genome Biology, Institute of Sericulture and Systems Biology, Southwest University, Beibei, Chongqing 400716, China; 2 Cancer Center, Medical Research Institute, Southwest University, Beibei, Chongqing 400716, China; 3 Engineering Research Center for Cancer Biomedical and Translational Medicine, Southwest University, Beibei, Chongqing 400716, China; 4 Chongqing Engineering and Technology Research Center for Silk Biomaterials and Regenerative Medicine, Southwest University, Beibei, Chongqing 400716, China

**Keywords:** antibiotics, tigecycline, cell cycle arrest, autophagy, mitochondrial translation, OxPhos

## Abstract

As an FDA-approved drug, glycylcycline tigecycline has been used to treat complicated microbial infections. However, recent studies in multiple hematologic and malignant solid tumors reveal that tigecycline treatment induces cell cycle arrest, apoptosis, autophagy and oxidative stress. In addition, tigecycline also inhibits mitochondrial oxidative phosphorylation, cell proliferation, migration, invasion and angiogenesis. Importantly, combinations of tigecycline with chemotherapeutic or targeted drugs such as venetoclax, doxorubicin, vincristine, paclitaxel, cisplatin, and imatinib, have shown to be promising strategies for cancer treatment. Mechanism of action studies reveal that tigecycline leads to the inhibition of mitochondrial translation possibly through interacting with mitochondrial ribosome. Meanwhile, this drug also interferes with several other cell pathways/targets including MYC, HIFs, PI3K/AKT or AMPK-mediated mTOR, cytoplasmic p21 ^CIP1/Waf1^, and Wnt/β-catenin signaling. These evidences indicate that antibiotic tigecycline is a promising drug for cancer treatment alone or in combination with other anticancer drugs. This review summarizes the biological function of tigecycline in the treatment of tumors and comprehensively discusses its mode of action.

## 1. Introduction

For many years, antibiotics have been used as the most important type of antibacterial agent for the treatment of bacterial infections. However, recent reports show that they have potential as drugs for cancer treatment. Firstly, some antibiotics can target DNA non-specifically and have been approved for use in the clinic to treat cancers, such as doxorubicin, daunorubicin, dactinomycin, idarubicin, mitoxantrone, and mitomycin C [1]. Secondly, since intestinal microbiota modulate the anticancer immune effects of some therapies, such as cyclophosphamide and anti-PD-L1 treatment, [2,3], modulation of the fecal microbiome with antibiotics may have an impact on the outcomes of cancer immunotherapy [4]. Thirdly, since mitochondria, which are highly similar to prokaryotic cells, are important for energy production and apoptosis regulation [5], antibiotics may target mitochondria to modulate cell survival and metabolism of tumor cells [6]. Lastly, antibiotics also have some other targets in the tumor cells, thereby affecting cell proliferation and survival. However, the detailed mechanistic aspects leading to pharmacological activity remain in part unknown.

Currently, many kinds of antibiotics including anthracyclines [7,8] (e.g., daunorubicin, doxorubicin, aclarubicin, epirubicin, idarubicin, valrubicin, and mitoxantrone), bleomycins (e.g., bleomycin A2) [9], mitomycins (e.g., mitomycin C) [10], dactinomycin [11], and macrolides antibiotics (e.g., azithromycin [12]) are being used as chemotherapeutical drugs to treat various malignant tumors. However, these antibiotics also exert toxicity for normal tissues. Recently, tetracyclines such as doxycycline, minocycline, and a chemically modified tetracycline, 6-deoxy, 6-demethyl, 4-de-dimethylamino tetracycline (CMT-3, COL-3) have been shown to have potential roles in several kinds of tumors [13,14,15]. For instance, a clinical pilot study shows that doxycycline, a Food and Drug Administration (FDA)-approved antibiotic, effectively reduces cancer stem cells, with a decrease of stem cell markers CD44 and ALDH1 expression, in early breast cancer patients [16]. Further study suggests that expression of stem cell markers including Oct4, Sox2, Nanog, and CD44 are also significantly downregulated and epithelial-to-mesenchymal transition (EMT) is also suppressed after doxycycline treatment in breast cancer [17]. Breast cancer related inflammation is also attenuated after doxycycline treatment, with decrease of plasma lysophosphatidate concentrations and inactivation of NF-κB [18].

**Figure 1:**
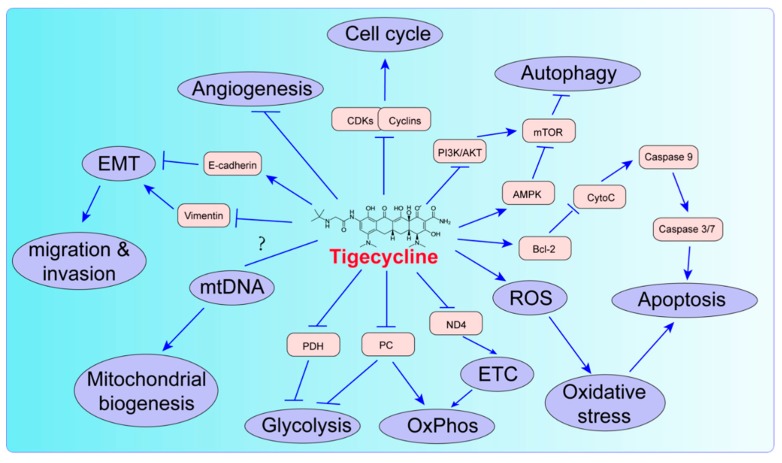
The biological effects of tigecycline in cancer cells. Tigecycline treatment induces decrease in OxPhos, dysfunction of mitochondria, inhibition of EMT and migration/invasion, suppression of angiogenesis, arrest of cell cycle, increase of ROS and oxidative stress/damages, promotion of intrinsic apoptois and activation of autophagy. CytoC, cytochrome C; EMT, epithelial–mesenchymal transition; ETC, electron transfer chain; mtDNA, mitochondrial DNA; ND4, NADH dehydrogenase subunit 4; OxPhos, oxidative phosphorylation; PC, pyruvate carboxylase; PDH, pyruvate dehydrogenase; ROS, reactive oxygen species.

Tigecycline (Figure 1) is a glycylcycline class antiobiotic, structurally similar to the tetracyclines. It inhibits protein translation by strong binding to the 30S ribosomal subunit, resulting in a blockage of the entry of charged aminoacyl-tRNAs into the A-site of the ribosome during prokaryotic translation in prokaryotes, hence preventing peptide elongation [19]. This drug was approved by the US FDA for the treatment of a range of bacterial infections in June 2005 [20]. It is highly active against gram-positive, anaerobic pathogens, and nearly all Gram-negative (except *Pseudomonas aeruginosa* and *Proteus* sp.). It has emerged as first line therapy for some serious systemic infections caused by multi-drug resistant (MDR) pathogens, e.g., carbapenem resistant *Enterobacteriae* [21].

Tigecycline has been firstly shown to have an anti-cancer effect in human acute myeloid leukemia (AML) via the inhibition of mitochondrial translation [22]. Then our group find that this drug also plays important roles in various solid tumors, such as gastric cancer [23], oral squamous cell carcinoma [24], melanoma [25], neuroblastoma [26], and glioma [27]. Meanwhile, other groups further reveal that tigecycline acts as a promising anti-cancer drug for many other kinds of cancers, including triple-negative breast cancer (TNBC), diffuse large B-cell lymphomas (DLBCLs), cervical squamous cell carcinoma, ovarian cancer, hepatocellular carcinoma (HCC), chronic myeloid leukemia (CML), acute lymphoblastic leukemia (ALL), lung cancer, prostate cancer and pancreatic cancer [6,28,29,30,31,32,33,34]. Recently, c-Abl-specific tyrosine kinase inhibitors (TKIs) in combination with tigecycline are also shown to be a promising strategy to treat patients with CML [35]. These evidences indicate that tigecycline may be a promising drug for cancer therapy.

In this article, we summarize the in vitro and in vivo studies referring to function of tigecycline and its mode of action in different kinds of tumors, in order to provide some clues for cancer treatment.

## 2. Biological Function of Tigecycline in Tumors

As shown in Figure 1, the main effects of tigecycline on cancer cells are inhibition of cell proliferation and aerobic metabolism as well as induction of cell cycle arrest and mitochondrial dysfunction. The IC50 of this drug on cancer cells is ranged from 5.8 to 51.4 μM (Table 1). In some kinds of cancer cells, tigecycline also induces autophagy and apoptosis, and inhibits angiogenesis and migration/invasion. Different phenotypes induced by this drug in different cancer cell lines are listed in Table 2.

### 2.1. Tigecycline Inhibits Mitochondrial Oxidative Phosphorylation

One of landmarks of cancer is altered cell metabolism. Many cancer cells rely on mitochondrial energy transduction and nutrient utilization pathways to enhance survival. As an antibiotic, tigecycline has the potential to target mitochondria, which is similar to prokaryotic cells. In Myc-driven lymphomas [37] and TEX leukemia cells treated with increasing concentrations of tigecycline over 4 months [38], abnormally swollen mitochondria with a decrease in the number and length of inner membrane cristae, and occasional perforation of the external membrane are observed after tigecycline treatment. Actually, tigecycline is shown to inhibit the mitochondrial oxidative phosphorylation (OxPhos) to restrict energy in various neoplasms, such as CML stem cells [35], ALL [34], non-small cell lung cancer (NSCLC) [28], ovarian cancer [39] and HCC [31].

Tigecycline suppresses ATP levels in RB1-deficient TNBC cells, which rely on the OxPhos, more effectively than in RB1-proficient cell lines, which does not rely on the OxPhos [29]. It significantly affects mitochondrial respiration and ATP levels in HCC cells [31]. Mitochondrial spare respiratory capacity (SRC) is the spare capacity between maximal respiration and mitochondrial basal. It can reflect the mitochondrial reserve capacity to produce energy. Tigecyline induces significantly diminution of SRC in OxPhos-diffuse large B-cell lymphoma (DLBCLs) [30]. In ovarian cancer cells, a significant decrease in basal and maximal mitochondrial respiration is observed after tigecycline treatment [39]. In TEX leukemia cells resistant to tigecycline, which are obtained from selected cells that are treated with increasing concentrations of tigecycline over 4 months, a reduced oxygen consumption is observed, compared with that of wild type cells. After removing tigecycline, mitochondrial mass and mitochondrial membrane potential (MMP) returned to wild type levels to re-establish aerobic metabolism [38]. Prolonged exposure of cells to ethidium bromide (EtBr) can produce mitochondrial DNA-depleted ρ0 ovarian cancer cells, which lack oxidative phosphorylation, pyruvate and uridine to maintain cell growth. Interestingly, the addition of pyruvate and uridine significantly rescues the tigecycline-induced apoptosis, suggesting that the dysfunction of OxPhos induced by this drug is essential for cell viability [39].

Further study reveals that primary CD34^+^ CML cells cultured with ^13^C 6-glucose significantly decrease the incorporation of ^13^C isotopes into citrate, glutamate and aspartate as well as the enzymatic activities of pyruvate dehydrogenase (PDH) and pyruvate carboxylase (PC) after tigecycline treatment, resulting in a robust decrease in glucose oxidation and extra cellular acidification [35]. Meanwhile, there is a significant decrease in the fraction of isotopologues with 2 or more ^13^C atoms following incubation with ^13^C 5-glutamine and ^13^C 16-palmitate, and the overall steady-state levels of aspartate, which plays an anaplerotic role for OxPhos in the leukemic stem cells (LSCs)-enriched population [35].

Besides, interaction of tigecycline with mitochondrial electron transport chain (ETC) complex I inhibitor rotenone and complex II inhibitor malonate cannot induce further apoptosis in tigecycline-treated ovarian cancer cells, compared to the cells treated with tigecycline alone, indicating that tigecycline may target ETC [39]. In Myc-driven lymphomas, tigecycline is shown to uncouple the ETC with 2,4-dinitrophenol (DNP) to prevent oxygen consumption and to reduce basal mitochondrial respiratory capacity, but do not induce significant decline in ATP levels [37]. Other studies also found other likely targets of tigecycline in mitochondria. For instance, in OxPhos-DLBCLs incubated 1 μM tigecycline for 24 h, the translation of mtDNA-encoded complex I subunit NADH dehydrogenase subunit 4 (ND4) is effectively inhibited, thus reducing the enzymatic activity of NADH dehydrogenase, but do not affect that of succinate dehydrogenase (SDH), a respiratory complex that is entirely encoded by the nuclear DNA [30]. Besides, this drug also reduces the content of ETC supercomplexes in DLBCL cell lines [30].

In addition, glycolysis is also downregulated after tigecycline treatment in primary CD34+ CML cells [35]. However, in TEX leukemia cells resistant to tigecycline, there is an enhanced rate of glycolysis. Furthermore, these cells also increase susceptibility to inhibitors of glycolysis and resistance to hypoxia. After medium with tigecycline was removed and normal medium was added and incubated for one week, glycolysis returned to the levels of wild-type cells. Re-treatment with tigecycline, the glycolytic phenotype of these cells was re-established [38]. A time-course study in ovarian cancer cells shows that the decease of mitochondrial respiration is the primary event, while the increased glycolysis flux is the secondary effect [39].

### 2.2. Tigecycline Induces Mitochondrial Oxidative Damage

As a natural byproduct of the normal metabolism, reactive oxygen species (ROS) are chemically reactive chemical species containing oxygen such as peroxides, superoxide, singlet oxygen, alpha-oxygen and hydroxyl radical [43]. Most of ROS are produced from mitochondrial energy metabolism, and further activate signaling pathways, in which some signaling are involved in cellular metabolic adaptation [44]. At high levels, ROS can oxidize proteins, lipids, and nucleic acids to damage or kill cancer cells [45]. Tigecycline also induces mitochondrial ROS, which was significantly more prominent in OxPhos-DLBCLs compared with BCR-DLBCLs with glycolytic phenotype [30]. Tigecycline significantly affects the mitochondrial function via decreasing MMP, and increasing levels of mitochondrial ROS levels, resulting in oxidative damages on DNA, proteins and lipids in HCC cells [31]. In ovarian cancer cells, an increase in cellular ROS levels and an oxidative DNA damage marker 8-hydrodeoxyguanosine (8-OHdG) were also observed after tigecycline treatment [39]. These results indicate that tigecycline induces high levels of ROS that cause oxidative damages.

### 2.3. Tigecycline Affects Mitochondrial Biogenesis

Increase of mitochondrial biogenesis is a crucial determinant of tumorigenesis [46]. Myc is a major regulator in promoting mitochondrial biogenesis in Burkitt’s lymphoma [47], resulting in increased mitochondrial mass, mitochondrial DNA (mtDNA) copy number and oxygen consumption rate. Burkitt’s lymphoma cells with Myc repression are resistant to tigecycline, compared with cells with functional Myc. These results indicate tigecycline’s effect is dependent on Myc and it may inhibit mitochondrial biogenesis [22]. Another study reveals that tigecycline treatment inhibits Wnt/β-catenin-mediated transcription of Myc in Hela ovarian cells [39]. However, lymphoma samples with more mitochondrial mass are more sensitive to this drug treatment and the levels of some mtRNAs such as AtpP6, CytB, Nd2, and Nd3 are increased after tigecycline treatment [22], indicating it promotes mitochondrial biogenesis. It is unusual that in Myc-driven lymphoma, mtDNA abundance remains unaltered after tigecycline treatment [37]. Cancer cells with different genetic backgrounds may have a different response to this drug treatment.

### 2.4. Tigecycline Induces Cell Cycle Arrest

Cell cycle is highly related with cell proliferation of tumor cells and regulators involved in are considered as promising targets for cancer therapy [48,49]. Our group demonstrate as tigecycline induces cell cycle arrest at G0/G1 phase in multiple malignant tumors, including melanoma [25], glioma [27], neuroblastoma [26], oral squamous cell carcinoma [24], and multiple myeloma [40]. Interestingly, different CDKs and cyclins are observed to have significant changes after tigecycline treatment. In oral squamous cell carcinoma and melanoma cells, CDK2 and cyclin E are significantly reduced after tigecycline treatment [24,25]. In glioma, CDK4 and cyclin D1 significantly decreased after 10 μM tigecycline treatment [27]. In neuroblastoma, CDK2, cyclin E1 and cyclin D1 significantly decreased after 10 μM tigecycline treatment [26]. In multiple myeloma, CDK2 and cyclin D1 decrease significantly after 10, 20 and 40μM tigecycline treatment [40]. However, a recent study shows that tigecycline also induces cell cycle arrest at G2/M phase in human ovarian cancers [39].

### 2.5. Tigecycline Induces Autophagy

Autophagy is an important degradation pathway that clears misfolded or damaged organelles, and also serves as an alternative programmed cell death excluding apoptosis in tumors [50,51]. In several kinds of tumors, tigecycline induces significant autophagy. In gastric cancer cells and multiple myeloma, AMPK pathway is activated via phosphorylation and subsequently inactivates mTOR and its downstream targets including p70S6K and 4E-BP1 after tigecycline treatment, thereby inducing cell autophagy [23,40]. Tigecycline treatment does not affect the phosphorylation of AKT in multiple myeloma. However, in CML, tigecycline induces autophagy by downregulating the PI3K-AKT-mTOR pathway [33]. Importantly, autophagy induced by tigecycline treatment seems to protect cancer cells from cellular stress. In multiple myeloma and CML, inhibition of autophagy with autophagy inhibitors like bafilomycin A1, chloroquine (CQ) and 3-methyladenine (3-MA) or ATG5 knockdown promotes the anti-cancer activity of this drug [33,40], suggesting that autophagy plays a cytoprotective role during tigecycline treatment.

### 2.6. Tigecycline Induces Apoptosis

Another type of programmed cell death is apoptosis, however, cancer cells prefer avoiding apoptosis induced by extracellular stress. Inducing apoptosis is considered as an efficient method to cure cancers [52,53]. From the previous studies, no obvious apoptosis is observed in gastric cancer [23], melanoma [25], glioma [27], neuroblastoma [26], oral squamous cell carcinoma [24], or multiple myeloma [40] cells treated with moderate concentrations (10 μM) of tigecycline. However, specific concentrations (50 μM in CML cells [33], 20 μM in ALL cells [34], 5 μM retinoblastoma cells [41], 1 μM in cervical squamous cell carcinoma cells [32], 10 μM in NSCLC cells [28], 2.5 μM in Myc-driven lymphoma cells [37], and 2.5 μM in ovarian cancer cells [39]) of tigecycline treatment induces significant intrinsic apoptosis by activating the intrinsic apoptosis pathway, with activation of BCL-2, release of cytochrome c, and cleavage of caspase-9/caspase-3/caspase-7. In Myc-driven lymphoma, level of mitochondrial membrane voltage-dependent anion channel (VDAC), an important regulator in metabolite exchange and apoptosis, is also slightly increased after tigecycline treatment [37].

### 2.7. Tigecycline Inhibits Migration/Invasion and Angiogenesis

Tumor metastasis is the reason for 90% of cancer-related mortality, and migration and invasion are emerged before metastasis [54,55]. Epithelial–mesenchymal transition (EMT) can convert polarized cancer cells into motile cells [56]. Tigecycline inhibits cell migration/invasion in melanoma cells via blocking the EMT by upregulating E-cadherin and downregulating vimentin, which are specific markers of the ETM [25]. However, these evidences should be further confirmed by in vivo experiments.

Tumor growth and metastasis is dependent on angiogenesis triggered by chemical signals secreted from cancer cells [57]. Several anti-angiogenesis drugs have been approved for use in cancer treatment, however, it is not enough to decrease anti-angiogenesis activity in single mechanism-based anti-angiogenic strategies [58]. Blood vessels may be remodeled by using other compensatory mechanisms. Therefore, exploring new drugs that target angiogenesis is necessary. In retinoblastoma, angiogenesis is inhibited by tigecycline treatment in both in vitro and in vivo experiments [41]. This result indicates that tigecycline may be a potential new drug for anti-angiogenesis.

## 3. Mode of Action of Tigecycline in Tumors

**Figure 2:**
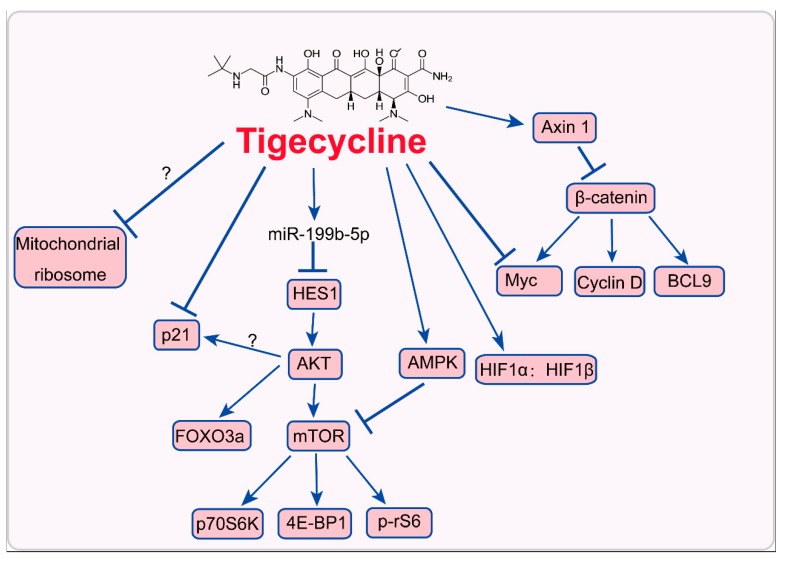
Mode of action of tigecycline in cancer cells. 4E-BP1, eukaryotic translation initiation factor 4E-binding protein 1; AMPK, AMP-activated protein kinase; BCL9, B-cell CLL/lymphoma 9; FOXO3a, forkhead box O3; HES1, hairy and enhancer of split homolog-1; mTOR, mammalian target of rapamycin; p70S6K, ribosomal protein S6 kinase; p-rS6, ribosomal S6 protein.

Since the exact targets of tigecycline have not been identified, it is dire need to unravel the mode of actions. Present studies show that tigecycline may interfere with proteins that translate in mitochondria or other signaling pathways such as Myc/HIFs, PI3K/AKT, AMPK signaling, p21^CIP1/Waf1^, and Wnt/β-catenin signaling pathways (Figure 2). These signaling pathways are essential for tumor development. Elucidating the mode of action of tigecycline would provide more clues for tigecycline used in cancer therapy.

### 3.1. Inhibition of Mitochondrial Translation

Since tigecycline is an antibiotic drug, it is assumed, mitochondria might represent the main target in eukaryotic cells. Mitochondrial translation is an important molecular biological process in cells. Although majority of mitochondrial proteins are encoded by the nuclear DNA, some important proteins are encoded by 16,569 b.p. mitochondrial DNA (mtDNA) and are translated via mitochondrial translation system [59]. In *Homo sapiens*, 22 tRNAs, 12S and 16S rRNAs, and 13 polypeptides including cytochrome c oxidase subunits I, II, and III, ATPase subunit 6, cytochrome b, and 8 other predicted peptides that constitute the central core of OxPhos complexes are encoded by mtDNA [60,61]. Mitochondrial translation is required for functional mitochondria, which is related to multiple biological processes, such as metabolic regulation, cell cycle progression, apoptosis, autophagy, oxidative response, angiogenesis, migration/invasion, and drug resistance during tumor development [62,63]. Recently, mitochondrial translation is shown as a promoter of tumor malignancy [22] and interference of this process is a promising method for the treatment of cancers [22,36,64].

Recently, tigecycline has been shown to impair mitochondrial translation in various blood malignancies and solid neoplasms such as AML [22,65], CML [33], diffuse large B-cell lymphoma [30], Myc-driven lymphomas [37], Burkitt’s lymphoma [22], renal cell carcinoma [42], and ovarian cancer [39]. In these cancer cells, tigecycline suppresses COX I and COX II, which are translated in mitochondria, as well as NADH: ubiquinone oxidoreductase subunit B8 (NDUFB8) protein levels, while COX IV (this protein is cytosolically translated), ubiquinol-cytochrome-c reductase core protein 2 (UQCRC2, COX III), succinate dehydrogenase complex flavoprotein subunit A (SDHA) and glucose-regulated protein, 78kDa (GRP78, HSPA5) remain unchanged [29,40]. Consistently, mRNA levels of COX I and COX II but not COX IV or GRP78, are upregulated after tigecycline treatment, further indicating that tigecycline inhibits mitochondrial translation [31,37]. In TEX leukemia cells resistant to tigecycline, very low levels of COX I and COX II (undetectable) were observed. In addition, upon removal of tigecycline from these cells, levels of COX I and COX II returned to the levels of wild-type cells [38]. These evidences suggest that tigecycline significantly impairs mitochondrial translation in cancer cells.

**Figure 3:**
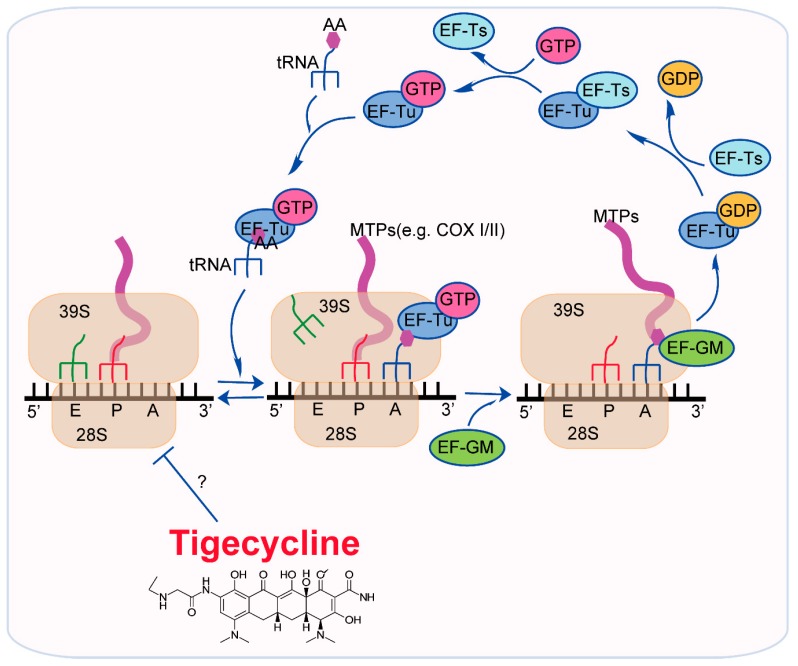
The effect of tigecycline on mitochondrial translation. Elongation factor EF-Tu binds to GTP to form a complex with tRNA and amino acid. Then this complex delivers (AA)-tRNA into ribosomal A site. Tigecycline may target the mitochondrial ribosome through inhibiting the EF-Tu/(AA)-tRNA/GTP complex delivery. AA, amino acid; EF-GM, elongation factor G, mitochondrial; EF-Ts, elongation factor Ts, mitochondrial; EF-Tu, elongation factor thermo unstable; GDP, guanosine-5’-diphosphate; GTP, guanosine-5’-triphosphate; MTPs, mitochondrial translated peptides.

However, the exact target of tigecycline in mitochondria has not been identified. As mitochondria are highly similar with prokaryotes, it is possible that tigecycline also inhibit mitochondria in eukaryotes via a similar molecular mechanism. Prokaryotes such as bacteria have small 30S and large 50S subunits, which together form a 70S mitoribosome [66]. Tigecycline binds to elongating ribosomes and suppresses the delivery of elongation factor thermo unstable (EF-Tu)⋅GTP aminoacyl (AA)-tRNA complex to the ribosomal A site [67]. Mechanically, tigecycline binds to 70S ribosome through interacting with 16S rRNA nucleobase C1054 which is located at the decoding site of the ribosome, thereby blocking the initial codon recognition step of tRNA accommodation [68]. In addition, tigecycline also binds to the 30S ribosomal subunit with its tail in an extended conformation and makes extensive interactions with the nucleotide C1054 [69]. Eukaryotic mitochondria have small 28S and large 39S subunits, which together forming a 55S mitoribosome [70]. Although eukaryotic mitochondrial ribosomes have some differences with prokaryotic ribosomes [71,72], they also have many similarities between each other [73,74]. EF-Tu (TUFM) is also one of tree mitochondrial elongation factors [the other two factors are elongation factor Ts (EF-Ts, TSFM) and elongation factor G, mitochondrial (EF-GM, GFM1) that participate into the elongation of mitochondrial translation (Figure 3) [75,76]. Mitochondrial EF-Tu recruits GTP and aminoacyl-tRNA to form a complex, which directs the tRNA to the acceptor site where the tRNA base pairs with the mRNA at the codon-anticodon site [77,78]. In AML and renal cell carcinoma, inhibition of mitochondrial translation by EF-Tu knockdown mimics the inhibitory effects of tigecycline via promoting mRNA expression and inhibiting protein expression of COX I and COX II, without changing COX IV protein or mRNA levels [22,42]. These evidences indicate that tigecycline may have a direct target in mitochondrial ribosome. However, the exact mode of action remains to be further elucidated.

### 3.2. Inhition of Myc and Activation of HIFs

Many cancers have genetic MYC activation, which can collaborate with hypoxia inducible factors (HIFs) to confer metabolic advantages to cancer cells for surviving in a hypoxic microenvironment [79,80]. Burkitt’s lymphoma cells with Myc repression are resistant to tigecycline, compared with cells with functional Myc. These results indicate that tigecycline may target Myc [22]. Meanwhile, MYC activation sensitizes MYC/BCL2 double-hit B cell lymphoma cells to inhibition of mitochondrial translation by the antibiotic tigecycline [81,82]. Another study shows that tigecycline treatment inhibits transcription of Myc mediated by Wnt/β-catenin signaling in Hela ovarian cells [39]. A previous study also indicates that tigecycline suppresses transcription of Myc through axin 1/Wnt/β-catenin pathway [32]. These evidences suggest Myc may be a possible downstream factor after tigecycline treatment in cancer cells. The expression of another major metabolic regulator, HIF1α is upregulated in TEX leukemia cells resistant to tigecycline. HIF1α: HIF1β transcription factor complex also enhances its binding to the promoters of its target genes, thereby promoting glycolysis. Upon removal of tigecycline, glycolysis returns to the levels of wild-type cells, but HIF1α remains unchanged [37]. Myc and HIFs are major regulators of aerobic glycolysis [83,84]. The effect of tigecycline on these two factors may be the reason for increased glycolysis observed in tigecycline-treated cancer cells. As increase of glycolysis is a subsequence of decreased OxPhos after tigecycline treatment, Myc and HIFs may also be a subsequence of decreased OxPhos and may not be a direct target of tigecycline. In fact, the exact mechanism is not so clear and need to be further explored.

### 3.3. Inhibition of PI3K/AKT-FOXO3a/mTOR Signaling

PI3K/AKT signaling is among major regulators of tumor progression [85]. In glioma, tigecycline treatment significantly increases the expression of miR-199b-5p, which target hairy and enhancer of split homolog-1 (HES1), a basic helix-loop-helix transcriptional repressor served as a downstream target of the Notch signaling pathway [86], thereby inactivating PI3K/AKT through reducing AKT phosphorylation at position Ser473 [27]. In neuroblastoma, this drug also decreases the phosphorylation of AKT at position Ser473, while not Thr308, thereby inhibiting the phosphorylation of its target FOXO3a at Ser253, while not Thr32 [26]. In CML and renal cell carcinoma, it downregulates PI3K/AKT, thus inhibits another target mTOR pathway [33,42]. These evidences indicate that tigecycline induces PI3K/AKT signaling via miR-199b-5p-HES1 axis, thereby weakening the phosphorylation of its target FOXO3a and suppressing mTOR signaling. In fact, PI3K/AKT signaling is also a major regulator of aerobic glycolysis [83,84], suggesting that this pathway may also be a consequence of metabolic alterations induced by tigecycline treatment.

### 3.4. Activation of AMPK-mTOR Signaling

AMP-activated protein kinase (AMPK) is an essential mediator in the maintenance of cellular energy homeostasis and cellular metabolism [87]. In gastric cancer cells, ovarian cancer and multiple myeloma, AMPK pathway is activated via phosphorylating at Thr172 and subsequently inactivates mTOR with dephosphorylation at Ser2448 and its downstream targets including p70S6K (dephosphorylation at Thr389), 4E-BP1 (dephosphorylation at Thr37/46) and p-rS6 (dephosphorylation at Thr37/46) after tigecycline treatment, thereby inducing cell autophagy or overcoming chemoresistance [23,39,40]. Tigecycline treatment does not affect the phosphorylation of AKT in multiple myeloma. Actually, it activates AMPK and inhibits mTOR signaling as a result of mitochondrial dysfunction [39]. May be tigecycline-induced decline of OxPhos and ATP levels activate AMPK to maintain energy homeostasis and to induce autophagy for the survival of cancer cells.

### 3.5. Inhibition of Cytoplasmic p21^CIP1/Waf1^

p21^CIP1/Waf1^, which is encoded by cdkn1a gene, was originally identified as a member of the cyclin-dependent kinases inhibitors (CKIs). Previously, nuclear p21 was determined as a tumor suppressor by inhibiting cyclin E-CDK2 activity and inducing cell cycle arrest at G1 phase [88]. However, in the cytoplasm, it acts pro-oncogenic properties regarding promotion of migration and proliferation as well as induction of apoptosis [89,90]. In melanoma samples, p21 expression is increased significantly, compared with that of intramucosal nevi [90,91] and high levels of p21 in melanoma protects against p53-mediated apoptosis [92]. Our previous study also reveals that both mRNA and protein levels of p21 is significant declined after tigecycline treatment in a dose- and time-dependent manner, and restoration of p21 in melanoma can rescue cell cycle arrest, cell proliferation inhibition, migration, and migration blockade induced by tigecycline treatment [25]. These results indicate that cytoplasmic p21 may be a target of tigecycline. However, how it induces downregulation of p21 remains unclear. Actually, Akt can phosphorylate p21 at threonine 145 to promote its cytoplasmic localization [93]. As shown above, tigecycline inhibits PI3K/AKT pathway signaling. It is possible that p21 decrease is a consequent event of PI3K/AKT pathway signaling inhibition induced by tigecycline.

### 3.6. Inhibition of Wnt/β-Catenin Signaling

The canonical Wnt/β-catenin signaling pathway is aberrantly activated in human cervical cancers and it can induce the transformation of HPV-immortalized keratinocytes as the second hit [94]. Recent study reveals that the expressions of cytoplasmic and nuclear β-catenin are significantly decreased after tigecycline treatment. Tigecycline can promote the expression of axin 1, a negative regulator of of β-catenin [95], which further suppresses Wnt/β-catenin-mediated transcription of Myc, Cyclin D and BCL9 in Hela cells. Pharmacological stabilization of β-catenin with LiCl or restoration of β-catenin through its overexpression via transfected plasmid, rescues the proliferation inhibition and apoptosis induced by tigecycline [32]. The data reveal that Wnt/β-catenin pathway is a downstream signaling that involves a tigecycline-induced effect. Since β-catenin is tightly associated with tumor invasiveness [96], this signaling may also play an important role in tigecycline-induced inhibition of migration and invasion. Actually, Wnt/β-Catenin and PI3K/Akt signaling crosstalk with each other through their common target GSK3β, and inhibition of PI3K/Akt signaling also suppresses GSK3β phosphorylation and β-catenin expression [97]. These evidences indicate that inhibition of Wnt/β-catenin signaling may also be a result of PI3K/Akt signaling suppression induced by tigecycline.

## 4. Effect of Tigecycline Combined with Other Anticancer Drugs

**Figure 4:**
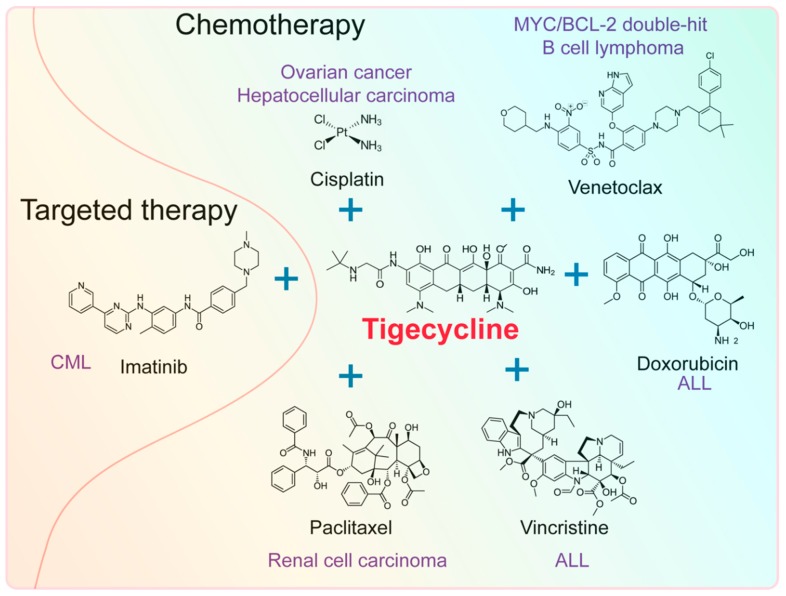
Tigecycline promotes susceptibility of tumors to chemotherapy and targeted therapy. ALL, acute lymphoblastic leukemia; CML, chronic myeloid leukemia.

Cancer cells can get drug resistance easily, which has become a major obstacle in the treatment of these diseases [98]. Recent studies reveal that tigecycline can enhance susceptibility of various tumors, especially hematological neoplasms to chemotherapy or targeted therapy (Figure 4), suggesting the suitability of the drug in combination with chemotherapy.

### 4.1. Effect of Tigecycline Combined with Chemotherapeutic Drugs

Tigecycline can enhance susceptibility of some hematological malignancies and solid tumors to chemotherapeutic drugs, such as venetoclax, doxorubicin, vincristine, paclitaxel, and cisplatin.

MYC/BCL double-hit lymphomas (DHL) are high-grade B cell lymphomas with MYC and BCL2 or BCL6 translocations. These lymphomas have a poor prognosis and are resistance to current front-line treatments in lymphomas, such as CHOP (cyclophosphamide, doxorubicin, vincristine, and prednisolone) chemotherapy and R-CHOP. BCL-2 inhibitor venetoclax show a most promising prospect to treat MYC/BCL-2 DHL. BCL-2 blocks the proapoptotic activity of MYC while remaining its capacity to promote cell proliferation. It is reasoned that compounds that exacerbate MYC-induced apoptosis would be likely to cooperate with venetoclax in killing DHL cells. Tigecycline is shown to induce significant apoptosis in MYC-driven lymphoma [37]. As expected, a pre-clinical study shows that tigecycline and venetoclax synergically inhibit MYC/BCL-2 DHL [81]. In another preclinical study, tigecycline dramatically improved the efficacy of doxorubicin and vincristine in ALL cell lines, primary samples and xenograft mouse model [34].

In addition to hematological malignancies, this drug also enhances susceptibility of solid tumors to chemotherapy. A recent clinical study shows that tigecycline is associated with a significant decrease in fibrinogen levels, which is a marker for malignant phenotype, following cytoreductive surgery (CRS) and hyperthermic intraperitoneal chemotherapy (HIPEC), compared to imipenem–cilastatin [99]. Importantly, tigecycline acts synergistically with cisplatin in ovarian cancer [39] and HCC [31] in vitro and in vivo. Besides, tigecycline combined with paclitaxel significantly enhances therapeutic efficacy of renal cell carcinoma in vitro and in vivo [42].

These results indicate that tigecycline can possibly be used as an auxiliary therapeutic drug for chemotherapy in both hematological malignancies and solid tumors.

### 4.2. Effect of Tigecycline Combined with Targeted Drugs

Tigecycline can also enhance susceptibility of some hematological malignancies target therapy, such as imatinib, a small molecule that specifically targets the TK domain in abl (the Abelson proto-oncogene), c-kit, and PDGF-R (platelet-derived growth factor receptor). For instance, tigecycline inhibits cell proliferation and induces apoptosis in KBM5 CML cells with T315I mutations (KBM5-STI cells), which is an imatinib-resistant genotype [33]. Combination treatment with imatinib and tigecycline selectively eradicate CML leukemic stem cells both in vitro and in a xenotransplantation model of human CML [35]. Rituximab is a monoclonal antibody that targets CD20 on the surface of the leukemia and lymphoma cells. A recent pre-clinical study shows that tigecycline combined with venetoclax promotes the primary response of MYC/BCL2 double-hit B cell lymphoma cells to rituximab-based chemoimmunotherapeutic regimens, also known as R-CHOP [81]. These results reveal that tigecycline can also be used as a promising drug during targeted therapy.

## 5. Concluding Remarks and Perspectives

In conclusion, tigecycline is a promising drug for cancer therapy alone or combined with chemotherapeutic or targeted drugs. As an FDA-proved drugs for infection treatment, this drug may be used in cancer treatment in the future. Although it also has some side effects, such as hypofibrinogenemia [100], mitochondrial dysfunction [101], chronic otitis [102], and gastrointestinal symptoms such as nausea, vomiting, and diarrhea [103], we cannot ignore its important potential promise in anticancer therapy [104]. A clinical study on tigecycline antibacterial activity shows that once daily high dose this drug, i.e., 200–400 mg (IV) × 1, then 100–200 mg (IV) q24 h, can effectively eliminate the multi-drug resistant (MDR) *Klebsiella Pneumoniae* in the urine in 5 days and *Enterobacter aerogenes* in 12 days without gastrointestinal or other side effects [105]. A phase 1 clinical study of tigecycline administered through intravenous injection daily 5 of 7 days for 14 days to patients with AML shows that the maximal evaluated dose is 350 mg/day and the maximal tolerated dose was 300 mg/day [65].

However, a phase 1 dose-escalation study of tigecycline administered in patients with AML by injecting intravenously daily 5 of 7 days for 2 weeks shows no significant pharmacodynamic changes or clinical responses [65]. Actually, tigecycline has a shorter half-life (9.5 h) in AML patients than that of noncancer patients [65]. This result means that it is easy to metabolize. Therefore, it is also dire need to develop a method or a new derivative to prolong the t^1/2^ of tigecycline. Fortunately, adding ascorbic acid and vitamin E into the formulation of tigecycline maintains the stability of this drug for at least 7 days [106]. It is valuable to test the new formulation of tigecycline in the next clinical study of tumors. Alternatively, the administration method of this drug would be changed into two times daily. A recent pre-clinical study in MYC/BCL2 double-hit B cell lymphoma shows that twice a day intraperitoneal injection of tigecycline (spaced by ~8 h) synergistically promotes the effect of venetoclax [81]. A multicenter, controlled, randomized clinical trial further reveals that the combination of piperacillin/tazobactam (4.5 g intravenously every 8 h) and tigecycline (50 mg intravenously every 12 h; loading dose 100 mg) is safe, well tolerated, and more effective than piperacillin/tazobactam alone in high-risk hematologic patients with cancer and febrile neutropeniae [107]. The results make the combination possibly to be considered as one of the first-line empiric antibiotic therapies. Although tumor progression is not investigated in this clinical study, the results also indicate that 50 mg tigecycline intravenously every 12 h is suitable for clinical use.

**Figure 5:**
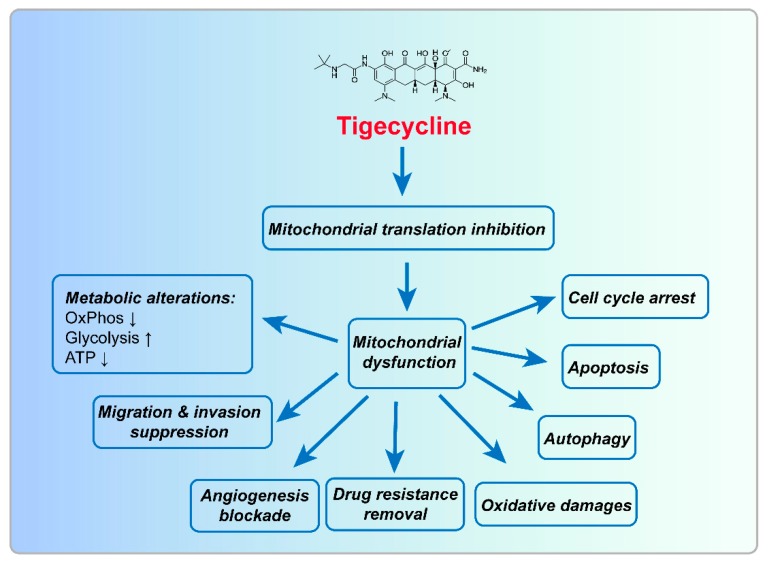
Hypothesis on the causality of phenotypes induced by tigecycline treatment in cancer cells.

The main mechanisms for tigecycline in cancer cells are inhibition of mitochondrial translation, PI3K/AKT- or AMPK-mediated mTOR signaling, cytoplasmic p21^CIP1/Waf1^ signaling and Wnt/β-catenin signaling, as well as activation of HIFs and interaction with Myc. However, the direct target of this drug in cancer cells has not been identified. It seems that tigecycline-induced inhibition of mitochondrial translation is the first event for the mechanism of tigecycline in cancer cells. Phenotypes including oxidative damages, autophagy, apoptosis, cell cycle arrest, migration/invasion suppression, angiogenesis blockade, drug resistance removal, and metabolic alterations including decreased OxPhos, upregulated glycolysis and decreased ATP levels observed in cancer cells after tigecycline treatment are consequences of mitochondrial dysfunction (Figure 5). In addition, every phenotype induced by tigecycline-mediated mitochondrial dysfunction may also highly interweaved with each other. If this hypothesis is true, the exact model of action of tigecycline-induced inhibition of mitochondrial translation is the main task to be explored further. Previous studies have revealed that mitochondrial ribosome seems to be a specific target of tigecycline, because deletion of EF-Tu mimics the effect of this drug in cancer cells [22,42]. However, the detailed mechanism should be further explored. As a drug, tigecycline may also have other direct targets that may also confer to the phenotypes induced by this drug.

## Figures and Tables

**Table 1:** IC50 of tigecycline in several types of tumor cells. Please note that direct comparison of the IC50 data is not straightforward since obtained in different conditions with different methods.

Cancer Type	Cell Line Name	IC50 (Treatment Time)	Methods	References
Melanoma	A375	7.24 μM (48 h)	MTT assay	[25]
	MV3	10.9 μM (48 h)		
Non-small cell lung cancer	A549	5.8 μM (14 d)	Colony formation	[28]
	PC19	8.7 μM (14 d)		
	H157	6.8 μM (14 d)		
	EBC-1	5.9 μM (14 d)		
CML	K562	51.4 μM (48 h)	Cell Counting Kit-8 assay	[33]
RB1/TP53-mutant human TNBC cells	BT549; MDA-MB-436; Du4475	average IC50 = 3 μM (72 h)	MTT assay	[29]
RB1-proficient/TP53-mutant TNBC cells	HCC38; Hs578t; MDA-MB-231	average IC50 ≈ 20 μM (72 h) *		
Mouse colon cancer	CT26	33 μM (72 h)	CyQuant direct cell proliferation assay	[36]

* This average IC50 of tigecycline in RB1-proficient/TP53-mutant TNBC cells was presumably calculated by using GraphPas Prism 6 according to the approximate inhibition rate from Figure 8D in the original article [29]. The authors only provided an information in the text that the average IC50 of tigecycline in RB1-proficient/TP53-mutant TNBC cells was more than 8 μM. CML: chronic myeloid leukemia; TNBC: triple-negative breast cancer.

**Table 2:** Different biological phenotypes and signaling pathways or molecular mechanisms induced by tigecycline in different cancer cells.

Classification	Cancer Type	Main Biological Phenotypes	Pathways or Molecular Mechanisms	References
Hematologic tumors	Myc-driven lymphomas	Abnormally swollen mitochondria, OxPhos↓, ETC↓, intrinsic apoptosis	Mitochondrial translation↓	[37]
	CML	OxPhos↓, autophagy, intrinsic apoptosis	PI3K-AKT-mTOR↓, mitochondrial translation↓	[33,35]
	ALL	OxPhos↓, oxidative damages	--	[34]
	DLBCLs	OxPhos↓, ETC↓, ROS↑	Mitochondrial translation↓	[30]
	AML	Abnormally swollen mitochondria, OxPhos↓	Mitochondrial translation↓, EF-Tu↓, HIFs↑	[22,38]
Solid tumors	NSCLC	OxPhos↓, intrinsic apoptosis, ROS↑, MMP↓, ATP levels↓	--	[28]
	Ovarian cancer	OxPhos↓, ETC↓, ROS↑, oxidative damage, cell cycle arrest at G2/M phase, intrinsic apoptosis	Mitochondrial translation↓, Myc↓	[6,39]
	HCC	ATP levels↓, OxPhos↓, ROS↑, oxidative damage	Mitochondrial translation↓	[31]
	RB1-deficient TNBC	ATP levels↓, OxPhos↓	Mitochondrial translation↓	[29]
	Melanoma	Cell cycle arrest at G0/G1 phase, migration/invasion↓	Cytoplasmic p21↓	[25]
	Glioma	Cell cycle arrest at G0/G1 phase	miR-199b-5p-HES1-AKT↑	[27]
	Neuroblastoma	Cell cycle arrest at G0/G1 phase	AKT-FOXO3a↓	[26]
	Oral squamous cell carcinoma	Cell cycle arrest at G0/G1 phase	CDK4-CCNE2↓	[24]
	Multiple myeloma	Cell cycle arrest at G0/G1 phase, autophagy	AMPK-mTOR↑	[40]
	Gastric cancer	Autophagy	AMPK-mTOR↑	[23]
	Retinoblastoma	Intrinsic apoptosis, oxidative damage, angiogenesis↓, ATP levels↓	--	[41]
	Cervical squamous cell carcinoma	Intrinsic apoptosis	Wnt/β-catenin↓	[32]
	Renal cell carcinoma	Intrinsic apoptosis	Mitochondrial translation↓, EF-Tu↓, PI3K/AKT-mTOR↓	[42]

‘↑’ represents activation or unregulation, and ‘↓’ represents inhibition or dowregulation. ALL: acute lymphoblastic leukemia; DLBCLs: diffuse large B-cell lymphomas; AML: acute myeloid leukemia; HCC: hepatocellular carcinoma.
